# Supplementary material for: Differential expression of groEL-1, incB, pyk-F, tal, hctA and omcB genes during Chlamydia trachomatis developmental cycle
Source: PLoS One. 2021 Apr 15;16(4):e0249358. doi: 10.1371/journal.pone.0249358 (PMC8049257; doi:10.1371/journal.pone.0249358)
Supplement: S1 Table — (DOCX) [file pone.0249358.s001.docx]

| Step | Process | Solution | Temperature | Time |
| --- | --- | --- | --- | --- |
| 1 | Fixation | 2% gluteraldehyde in EMEM | 24^o^C | 30 min |
| 2 | Wash | EMEM | 24^o^C | 5 min |
| 3 | Wash | EMEM | 24^o^C | 5 min |
| 4 | Wash | Sodium cacodylate buffer | 24^o^C | 5 min |
| 5 | Post- fixation | 1% osmium tetroxide^a^ | 24^o^C | 1hour |
| 6 | Wash | Sodium cacodylate buffer | 24^o^C | 5 min |
| 7 | Wash | Sodium cacodylate buffer | 24^o^C | 5 min |
| 8 | Dehydration | 50% ethanol | 24^o^C | 10 min |
| 9 | Dehydration | 70% ethanol | 24^o^C | 10 min |
| 10 | Dehydration | 90% ethanol | 24^o^C | 10 min |
| 11 | Dehydration | 100% ethanol | 24^o^C | 10 min |
| 12 | Dehydration | 100% ethanol | 24^o^C | 10 min |
| 13 | Dehydration | 100% ethanol | 24^o^C | 10 min |
| 14 | Infiltration | Ethanol : Spurr resin (1:1)^b^ | 24^o^C | 30 min |
| 15 | Infiltration | Spurr resin^b^ | 60^o^C | 1 hour |
| 16 | Infiltration | Spurr resin^b^ | 60^o^C | 1 hour |
| 17 | Embedding | Spurr resin | 60^o^C | 8 hours |

**S1 Table 1. Processing schedule for TEM**

^a^ protected from light

^b^ procedure carried out uncovered to allow polypropylene to evaporate
