# Supplementary material for: Differential expression of groEL-1, incB, pyk-F, tal, hctA and omcB genes during Chlamydia trachomatis developmental cycle
Source: PLoS One. 2021 Apr 15;16(4):e0249358. doi: 10.1371/journal.pone.0249358 (PMC8049257; doi:10.1371/journal.pone.0249358)
Supplement: S2 Table — (DOCX) [file pone.0249358.s002.docx]

**S2 Table. Mean fold changes for six chlamydial genes in HaCaT cells**

| **A.** |  |  |  |
| --- | --- | --- | --- |
| ***groEL-1* (fold difference mean)** | | | |
| **Time (hrs) post infection** | **L2** | **E** | **US151** |
| **2** | 96,89 | 40,18 | 8,27 |
| **12** | 72,17 | 37,37 | 6,8 |
| **24** | 32,7 | 7,43 | 3,4 |
| **36** | 41,56 | 5,06 | 7,61 |
| **48** | 1,62 | 19,45 | 2,64 |

**B.**

| ***incB* (fold difference mean)** | | | |
| --- | --- | --- | --- |
| **Time (hrs) post infection** | **L2** | **E** | **US151** |
| **2** | 37,6 | 15,35 | 3,33 |
| **12** | 0 | 36,46 | 2,98 |
| **24** | 1,31 | 2,09 | 1,42 |
| **36** | 3,84 | 0 | 2,38 |
| **48** | 2,66 | 7,01 | 6,17 |

**C.**

| ***Pyk-F* (fold difference mean)** | | | |
| --- | --- | --- | --- |
| **Time (hrs) post infection** | **L2** | **E** | **US151** |
| **2** | 194,19 | 19,05 | 99,7 |
| **12** | 32,81 | 9,21 | 22,31 |
| **24** | 1,84 | 2,41 | 2,17 |
| **36** | 4,32 | 0 | 2,62 |
| **48** | 0,55 | 6,83 | 1,22 |

**D.**

| ***tal* (fold difference mean)** | | | |
| --- | --- | --- | --- |
| **Time (hrs) post infection** | **L2** | **E** | **US151** |
| **2** | 0 | 6,57 | 0,82 |
| **12** | 0 | 7,43 | 0 |
| **24** | 1,13 | 1,53 | 0 |
| **36** | 0 | 0 | 0 |
| **48** | 0 | 0 | 0,75 |

**E.**

| ***hctA* (fold difference mean)** | | | |
| --- | --- | --- | --- |
| **Time (hrs) post infection** | **L2** | **E** | **US151** |
| **2** | 3,6 | 1,32 | 1,35 |
| **12** | 2,4 | 0 | 1,04 |
| **24** | 1,32 | 1,34 | 1,03 |
| **36** | 1,63 | 0 | 0,95 |
| **48** | 0 | 0,57 | 0 |

**F.**

| ***omcB* (fold difference mean)** | | | |
| --- | --- | --- | --- |
| **Time (hrs) post infection** | **L2** | **E** | **US151** |
| **2** | 7,74 | 3,98 | 0,51 |
| **12** | 0 | 3,54 | 0 |
| **24** | 6,47 | 0 | 0 |
| **36** | 8,5 | 0 | 0 |
| **48** | 0 | 1,23 | 1,14 |
