# Supplementary material for: Differential expression of groEL-1, incB, pyk-F, tal, hctA and omcB genes during Chlamydia trachomatis developmental cycle
Source: PLoS One. 2021 Apr 15;16(4):e0249358. doi: 10.1371/journal.pone.0249358 (PMC8049257; doi:10.1371/journal.pone.0249358)
Supplement: Attachment — Submitted filename: Response to reviewers.docx [file pone.0249358.s003.docx]

March 9, 2021

Dear Dr. Michael F Minnick

Re: Revisions to manuscript ID: PONE-D-21-02174

We thank you for this opportunity to improve our manuscript “Differential gene expression by Chlamydia trachomatis in keratinocytes at different temperatures”. The comments from reviewers were very constructive and enabled us to greatly improve the manuscript. May I kindly request that the following amendments be made on my existing funding statement; This study was funded by grants from the National Research Foundation (SFH14061869970) and The University of Kwa-Zulu Natal College of Health Sciences. GFM is funded with a postdoctoral fellowship from the National Research Foundation (SFP180507326699). In the following section are our point‐by‐point responses to each of the comments of the reviewers.

**Editors comments:**

1. Please ensure that your manuscript meets PLOS ONE's style requirements, including those for file naming. The PLOS ONE style templates can be found at <https://journals.plos.org/plosone/s/file?id=wjVg/PLOSOne_formatting_sample_main_body.pdf>  and<https://journals.plos.org/plosone/s/file?id=ba62/PLOSOne_formatting_sample_title_authors_affiliations.pdf>

**Response: Manuscript has been formatted according to PLOS ONE's style requirements as suggested.**

2. Thank you for stating the following in the Acknowledgments Section of your manuscript:

'..This study was funded by grants from the National Research Foundation (SFH14061869970) and The University of Kwa-Zulu Natal College of Health Sciences. GFM is funded with a postdoctoral fellowship from the National Research Foundation (SFP180507326699).'

We note that you have provided funding information that is not currently declared in your Funding Statement. However, funding information should not appear in the Acknowledgments section or other areas of your manuscript. We will only publish funding information present in the Funding Statement section of the online submission form.

a. Please remove any funding-related text from the manuscript and let us know how you would like to update your Funding Statement. Currently, your Funding Statement reads as follows:

'Author: G.F Mzobe
Funder: National Research Foundation ([www.nrf.ac.za](http://www.nrf.ac.za/))
Grant number: SFH14061869970

The funders had no role in study design, data collection and analysis, decision to publish, or preparation of the manuscript.'

**Response: Funding-related text has been removed from the manuscript as suggested.**

b. Please include your amended statements within your cover letter; we will change the online submission form on your behalf.

**Response: This has been added in the cover letter as suggested**

2. Please amend either the title on the online submission form (via Edit Submission) or the title in the manuscript so that they are identical.

**Response: Title has been amended on the online submission form as follows; “*Differential expression of groEL-1, incB, pyk-F, tal, hctA* and *omcB* *genes during Chlamydia trachomatis* *developmental cycle*”.**

4. Please include a separate caption for each figure in your manuscript.

**Response: A separate caption for each figure has been included.**

5. Please include your table 1 as part of your main manuscript and remove the individual file. Please note that supplementary tables should remain as separate "supporting information" files.

**Response: Table 1 has been included in the manuscript.**

6. Please include captions for your Supporting Information files at the end of your manuscript, and update any in-text citations to match accordingly. Please see our Supporting Information guidelines for more information: http://journals.plos.org/plosone/s/supporting-information

**Response: Captions for the supporting information files have been added at the end of the manuscript**

Additional Editor Comments:

While there is certainly value in examining alternate host cells and various serovars of Chlamydia for analysis of expression patterns, the manuscript contains a number of major issues that must be addressed before it is suitable for publication.

The two reviewers have disparate evaluations of your manuscript and both are experts in the field. Unfortunately, my analysis of the paper falls more in line with reviewer 1. My major concerns are as follows:

1. There are missing data in both figures without any explanations,

**Response: We agree with the editor and the reviewers that some data was missing in figures without any explanation, we have since provided detailed explanation in the text and figure legends in the manuscript. For figure1: Some of the genes in response to serovar infections could not be expressed at 2 (strain L2: *tal*), 12 (strain L2: *inc B*, *tal*, *omcB*; serovar E: *hctA*; US151: *tal*, *omcB*), 24 (serovar E: *omcB*; US151: *tal*, *omcB*), 36 (serovar E: *incB*, *pyk-F*, *tal*, *hctA*, *omcB*; US151: *tal*, *omcB*), 48 hours post-infection (strain L2: *tal*, *hctA*, *omcB*; serovar E: *tal*; US151: *htcA*) using qPCR. For figure 2: Gene expression profile of certain genes in HeLa cells could not be detect at 2 (strain L2: *pyk-F*, *hctA*; US151: *pyk-F*, *htcA*) and 12 hours (L2: *hctA*; US151:*hctA*) post infection using qPCR, which is in keeping with published data.**

2. Lack of statistical analyses for comparing data

**Response: We have consulted with a qualified senior statistician in our group and she shared the same sentiments that p values can only be produced when there is a hypothesis to be tested. She added that, statistically, p value gives us the probability of obtaining data as extreme as or more extreme than observed given the null hypothesis. On these basis, it mean that the p value and null hypothesis are tied together. Our study is descriptive with limited data points and we don’t intend to produce any inferential statistics.**

3. inconsistencies between the data in your study and to previously-published results on temporal gene expression in Chlamydia (see citation [10]). For example, the late-cycle genes examined (hctA, omcB) are only modestly expressed at all the time points, and a mid-cycle gene (pykF) peaked at 2 hpi.

**Response: the editor and the reviewers are correct that our data differs with previous studies. We also observed this difference and we then validated our findings and technique by infecting both HeLa and HaCaT cell line using LGV serovars. Our findings for HeLa cells were consistent with previous studies but not with our HaCaT derived findings. This may be due to number of reasons: (1) HaCaT cells are untreated skin cells that were infected with serovars with low passage in our study compared to previous studies that used human epithelial cell lines transformed by viral oncogenes and treated with cycloheximide or DEAE-dextran to grow Chlamydia in the presence of serovars with high passage number. (2) HeLa cell have been suggested to have poor differentiation capabilities and have impaired ability to stratify properly as previously observed by (**[**Blanton et al., 1991**](https://www.frontiersin.org/articles/10.3389/fcimb.2017.00438/full#B7)**;** [**Tsunenaga et al., 1994**](https://www.frontiersin.org/articles/10.3389/fcimb.2017.00438/full#B70)**). In agreement with Joubert and Sturm (2011), Noguiera et al., (2017) also argues that the monolayer of HaCaT cells is able to support chlamydial growth and supports their use when assessing the impact of the stratified architecture and cell differentiation on *Chlamydia trachomatis* infection.**

*Blanton, R. A., Perez-Reyes, N., Merrick, D. T., and McDougall, J. K. (1991). Epithelial cells immortalized by human papillomaviruses have premalignant characteristics in organotypic culture. Am. J. Pathol. 138, 673–685.*

*Tsunenaga, M., Kohno, Y., Horii, I., Yasumoto, S., Huh, N. H., Tachikawa, T., et al. (1994). Growth and differentiation properties of normal and transformed human keratinocytes in organotypic culture. Jpn. J. Cancer Res. 85, 238–244. doi: 10.1111/j.1349-7006.1994.tb02088.x*

*Joubert, B. C., and Sturm, A. W. (2011). Differences in Chlamydia trachomatis growth rates in human keratinocytes among lymphogranuloma venereum reference strains and clinical isolates. J. Med. Microbiol. 60, 1565–1569. doi: 10.1099/jmm.0.032169-0*

*Nogueira, A.T., K.M. Braun, and R.A. Carabeo, Characterization of the Growth of Chlamydia trachomatis in In Vitro-Generated Stratified Epithelium. Front Cell Infect Microbiol, 2017. 7: p. 438.*

**Reviewer #1:**

This manuscript examines developmental gene regulation of 4 Chlamydia trachomatis strains in an immortalized human keratinocyte cell line. Six chlamydial genes shown in multiple studies to be differentially expressed throughout the developmental cycle are used to analyze temporal gene expression at 5 different time points. There may be some utility in this study for those studying chlamydial strains infected keratinocytes. However, the results really don't follow any patterns established in the literature and do not appear to be consistent between Figs 1 and 2. I have only a few comments for consideration.

1. *line 87. The HaCaT cell line may be useful in specific instances but I doubt that it is "the most appropriate cell line" to use. Statements of significance or priority should be minimized.*

**Response: We have since downplayed the statement and changed to “HaCaT cells are one of the cell lines than can be used to interrogate cell differentiation, growth kinetics, and gene expression of *C. trachomatis* infection” line 98 of the manuscript**

2. *There are no statistics for Figures 1 or 2. The legend of Fig 1 states that the standard deviations were incorporated into the fold-difference calculations. Perhaps a more qualified statistician might be needed to evaluate this but the statement on line 163 that " could not produce any p-values due to limited data points"*

**Response: We have consulted with a qualified senior statistician in our group and she shared the same sentiments that p values can only be produced when there is a hypothesis to be tested. She added that, statistically, p value gives us the probability of obtaining data as extreme as or more extreme than observed given the null hypothesis. On these basis, it mean that the p value and null hypothesis are tied together. Our study is descriptive with limited data points and we don’t intend to produce any inferential statistics.**

3. *There are missing data points in both figures with no explanation. There are no discernable patterns in Fig. 1. Indeed, in the bottom 3 panels some genes did not change at all. The differences do not really seem congruent with Fig. 2.*

**Response: We have since provided detailed explanation on missing data points in the text and figure legends in the manuscript. For Fig. 1: Some of the genes in response to serovar infections could not be expressed at 2 (strain L2: *tal*), 12 (strain L2: *inc B*, *tal*, *omcB*; serovar E: *hctA*; US151: *tal*, *omcB*), 24 (serovar E: *omcB*; US151: *tal*, *omcB*), 36 (serovar E: *incB*, *pyk-F*, *tal*, *hctA*, *omcB*; US151: *tal*, *omcB*), 48 hours post-infection (strain L2: *tal*, *hctA*, *omcB*; serovar E: *tal*; US151: *htcA*) using qPCR. For figure 2: Gene expression profile of certain genes in HeLa cells could not be detect at 2 (strain L2: *pyk-F*, *hctA*; US151: *pyk-F*, *htcA*) and 12 hours (L2: *hctA*; US151:*hctA*) post infection using qPCR, which is in keeping with published data.**

**Fig. 1 shows gene expression in HaCaT cells only, whereas Fig.2 compares expression of 3 genes (one for each chlamydial developmental stage) in HaCaT (L2: maroon; US151: blue) Vs HeLa (L2: orange; US151: purple) cells. Patterns for gene expression levels in HaCaT cells (Fig. 2) are in agreement with the patterns shown in Fig.1. However, the patterns observed in HeLa cells are in keeping with previously published data and not our HaCaT cell derived findings.**

4. *Caution should be exercised in interpreting results from the earliest time points. Carryover mRNA is known to be present in EBs and not due to early transcription.*

**Response: we note the reviewer’s valuable comment and we have added this to our limitation section in the discussion and reads as follows: “Taken together, the observed differences in transcriptional expression of chlamydial genes between reference strain (L2 434) and clinical isolates (US151 and serovar E) and inconsistency with published data may not be attributed exclusively to the type of model or the MOI used, but to several other factors. These include carryover mRNA that may be present in EBs, high-passage numbers, different regulatory systems for gene expression in HaCaT compared to HeLa cell lines, and the origin (site of infection) of the isolated strain.”**

5. *line 200. "G (E) strain" What is this? Does G represent genital. If so, please spell it out and identify serovars as such.*

**Response: "G (E) strain" has been changed to “the genital biovar (serovar E)”. This has also been corrected throughout the manuscript.**

6. *Urogenital strains are generally slower growing than LGV strains. Some of the LGV isolates seem to have different patterns of expression. A better way to get at this would be one step growth curves plotting numbers of progeny IFUs over time.*

**Response: this study is an ancillary of a parent study (Joubert and Sturm, 2011) that has done the *growth curves plotting numbers of progeny IFUs over time***

**Joubert, B. C., and Sturm, A. W. (2011). Differences in *Chlamydia trachomatis* growth rates in human keratinocytes among lymphogranuloma venereum reference strains and clinical isolates. *J. Med. Microbiol*. 60, 1565–1569. doi: 10.1099/jmm.0.032169-0**

7. *Fig 3. Scale bars are unreadable and it appears that different magnifications are shown. It would be easier to evaluate if all images were at a similar magnification.*

**Response: Scale bars have been made visible. Magnification is similar across each time point.**

8. *The manuscript would benefit from editing for English grammar.*

**Response: this has been resolve throughout the manuscript**

**Reviewer #2:**

The manuscript by Mzobe and colleagues addresses chlamydial gene expression in keratinocytes. The work is thorough and conducted well. The conclusions, though only modestly incremental, are sufficiently significant to warrant publication. The electron microscopy images will be particularly useful to readers.

I have no significant criticisms of the study, the following are minor changes that should be addressed by the authors:

1. *Line 35: keratinocytes should be plural*

**Response: “Keratinocyte” has been changed to “keratinocytes”**

2. *Line 45: Use Chlamydia spp., and italicize.*

**Response: “Chlamydia” was changed to “*Chlamydia spp.”***

3. *Line 49: “can occur either as…” should be changed.*

**Response: “can occur either as” to “In the extracellular phase, the organism manifests as the elementary body (EB)”**

4. *Line 62: indicate which inc*

**Response: Specified inc protein as *incB***

5. *Line 104: “strains” should be used in place of “serovars”. Same with line 120*

**Response: “serovars” changed to “strains”**

6. *Line 198: Why use biovar here instead of serovar?*

**Response: *a combination of serovar can be termed biovar***

7. *Figure 2: The color for US151 should be changed. The distinction will not be visible to most readers.*

**Response: The colour for US151 in figure 2 has been changed from red to purple.**

8. *There is some funniness in the references that should be worked out.*

**Response: References have been sorted out**

Yours sincerely,

Gugulethu Mzobe, PhD

**PostDoctoral Fellow: CENTRE FOR THE AIDS PROGRAMME OF RESEARCH IN SOUTH AFRICA**

**T:** +27 (0)31 655 05728   **F:** +27 (0)31 260 4566
